# Preemptive kidney transplantation: why, when, and how?

**DOI:** 10.1590/2175-8239-JBN-2022-0085en

**Published:** 2022-09-30

**Authors:** Ana Flávia Moura, José A. Moura-Neto, Lucio R. Requião-Moura, Álvaro Pacheco-Silva

**Affiliations:** 1 Escola Bahiana de Medicina e Saúde Pública, Departamento de Clínica Médica, Salvador, BA, Brasil.; 2 Universidade Federal de São Paulo, Escola Paulista de Medicina, Departamento de Medicina, Divisão de Nefrologia, São Paulo, SP, Brasil.; 3 Hospital Israelita Albert Einstein, Unidade de Transplante Renal, São Paulo, SP, Brasil.

**Keywords:** Preemptive Kidney Transplantation, Renal Insufficiency, Chronic, Renal Replacement Therapy, Transplante de Rim Preemptivo, Insuficiência Renal Crônica, Terapia de Substituição Renal

## Abstract

Among renal replacement therapies, preemptive kidney transplantation (PKT) presents the best clinical, social, and economic results. However, it is still infrequently chosen as first therapy for patients with irreversible kidney failure. Initiatives in different parts of the world were developed to identify the reasons why PKT is still not widely used and to facilitate the access of patients with end-stage kidney disease to the advantages associated with it. This article addresses the main advantages and difficulties of PKT and discusses when it should be indicated and how to prepare potential recipients for PKT.

## Resumo

Entre as terapias renais substitutivas, o transplante renal preemptivo (TRP) apresenta os melhores resultados clínicos, sociais e econômicos. No entanto, ainda é raramente escolhido como primeira terapia para pacientes com falência renal irreversível. Foram desenvolvidas iniciativas em diferentes partes do mundo para identificar as razões pelas quais o TRP ainda não é amplamente utilizado e para facilitar o acesso de pacientes com doença renal em estágio terminal às vantagens associadas ao mesmo. Este artigo aborda as principais vantagens e dificuldades do TRP e discute quando ele deve ser indicado e como preparar potenciais receptores para o TRP.

## Preemptive Transplantation: What Is It?

Preemptive kidney transplantation (PKT), defined as a kidney transplant performed before the start of maintenance dialysis, may be considered the optimal therapy for most patients with end-stage kidney disease (ESKD)^1^. One of the most significant advantages of PKT is the avoidance, or at least delay of dialysis-related risks^2^. It also offers better post-transplant clinical outcomes^3^ and lower medium- and long-term financial costs^4,5^.

However, PKT is not commonly performed around the world. For instance, in the United States (US), only 9.3% (14,620 out of 157,073) of all kidney transplants performed between 2000 and 2018 were in the preemptive modality^6^. The rate of PKT is even lower in other countries, such as Spain (5%), Uruguay (5.4%), or Indonesia (2.7%)^7^. Although the Transplantation Registry in Brazil does not officially count PKT, data from Hospital do Rim – the largest kidney transplant center in Brazil and the world – indicate that 16.67% (234 of 1,404) of all living donor kidney transplants between 2011 and 2016 were performed preemptively^8^.

There are several reasons for the low PKT rate, some of which are quite complex. Policies for allocating organs, ethical issues, patient and care team education, late referral to the nephrologist, and a time- and energy-consuming donor evaluation process are some of the barriers to PKT^2,3,9^.

In 2007, the National Kidney Foundation convened a Kidney Disease Outcomes Quality Initiative (NKF/KDOQI) conference to discuss a “Transplant First” approach as a primary goal in the care of ESKD patients. This initiative aimed to identify key difficulties for PKT and what could be done to overcome these barriers^10^. Similar initiatives have been observed in other countries. Their goal is to offer the clinical advantages of PKT to a greater number of patients, and its economic and social benefits, beyond reducing the overall waiting list for kidney transplantation.

The present narrative review aims to discuss advantages related to PKT, why it is infrequently performed, when is the best moment to perform it, and how patients should be prepared for this therapy.

## Why?

PKT has several advantages over transplantation performed after the start of dialysis. These advantages are independent of kidney transplant recipients’ characteristics, such as age and gender^11^. From a clinical point of view, PKT provides a lower risk of allograft failure and acute rejection, higher allograft survival, and less need for pre-transplant blood transfusions – since dialysis patients tend to have lower hemoglobin levels than non-dialysis ESKD patients under conservative care^2,3,12–14^.

A retrospective study with data from the U.S. Renal Data System evaluated 8,481 patients who received a living donor kidney transplant. When compared with living donor kidney transplantation after dialysis, PKT with living donors was associated with a 52% lower risk of allograft failure during the first year after transplantation (rate ratio 0.48; p = 0.002), an 82% decrease in the second year (rate ratio, 0.18; p = 0.001), and an 86% decrease in subsequent years (rate ratio, 0.14; p = 0.001)^13^.

In addition, PKT patients are spared of the potential risks associated with dialysis therapy, such as catheter-related infection, cardiovascular adverse effects such as left ventricular hypertrophy, hypertension, and intradialytic complications such as hypotension^1^.

Despite all the clinical benefits, it is unclear what the reasons for these advantages are. A 2004 study compared glomerular filtration rate (GFR) six months after transplantation and the subsequent rate of loss of renal function in 34,997 non-PKT with 5,966 PKT recipients. The mean GFR after six months of transplantation was similar among recipients from non-PKT (49.2 ± 14.7 mL/min/1.73 m^2^) and PKT (49.5 ± 5.7 mL/min/1.73 m^2^). Although PKT showed indeed a ‘modestly’ slower annual decline in GFR than non-PKT, the superior allograft survival of PKT could not be justified by preservation of native renal function or by differences in the rate of loss of renal function in that study^15^.

Of note, a few recent studies from France and Spain that evaluated PKT with deceased donors only showed no difference in clinical outcomes, such as life expectancy with a functioning graft^16^, early allograft loss, delayed graft function, and acute rejection^17^. Despite quite similar results, these studies paradoxically reached opposite conclusions. While the Spanish study highlighted that PKT provides better quality of life, lower costs, and comparable clinical outcomes^17^, the French study questioned the use of deceased donors for PKT due to the consequential increase in the waiting list^16^.

The advantages of PKT transcend the clinical aspects and extend to the social and economic levels. On a social level, patients are better able to continue their usual activities, such as exercise and work^9^. They also retain their Independence and freedom to comply with their previous routine, which cannot be maintained once dialysis is started.

Conversely, a recent study failed to show improvement in quality-of-life and mental satisfaction after PKT compared with non-PKT. Despite significant limitations, such as its retrospective nature, small sample size (n = 88), and the fact that it was a single-center study, the study results were somehow unexpected. However, the authors explain that kidney transplants may not dramatically improve the quality of life if the patient had not experienced the burden of dialysis. Contrarily, patients can even feel uncomfortable after transplantation due to the regular intake of immunosuppressive drugs. Non-PKT patients may have improved quality of life because they had previous experience with dialysis^18^.

From an economic perspective, PKT might have higher initial costs than dialysis due to surgical procedure, hospitalization, and immunosuppressive drug therapy. However, there is a medium-term compensation for these costs, as PKT has a smaller impact on annual expenses per patient when compared to expenses with dialysis in the long run^4,5^. A 2018 US study that compared costs of kidney transplantation with dialysis showed that the predicted costs per quality-adjusted life-years over ten years was US$ 39,939 for HLA-compatible living donor transplantation compared with US$ 72,476 for dialysis^5^.

However, even with clinical, social, and economic advantages, PKT is still rarely performed in the world^6,7^. Some barriers, mainly related to ethical issues and patient education, make it difficult to increase the use of PKT.

## Why Not?

Some aspects are relevant when choosing the most appropriate therapy for ESKD patients. There are ethical issues that must be addressed, mainly related to deceased donor PKT. In these cases, government and public policy makers seek to provide as equal chances as possible to patients on the waiting list through equitable organ allocation.

To do so, several ethical principles are considered: equity, priority (balance between waiting time on the list, disease severity, among other criteria), medical urgency, efficiency, utility, therapeutic outcomes, autonomy, and responsibility. The great challenge lies in balancing these principles, respecting the hierarchy of importance, without disregarding any principle. With this goal in mind, several ethical models can be adopted, depending on the prioritized criteria.

The social utility model (utilitarianism theory), for example, gives priority to the patients most useful to the community^19^. The prioritarianism theory, on the other hand, defends the prioritization of the most seriously ill patients (worst-off)^20^, while the beneficiality model considers criteria such as longer life expectancy and greater number of lives saved^21^. Perhaps the best known of all ethical theories in medicine, the equity model advocates equal chances for all patients^22^. However, some authors criticize this model, arguing that this proposal is impossible to apply in practice^23^.

Following the justice-based system, many theories prioritize impartial criteria (time on waiting list and allocation by lottery)^21,24^. Waiting list time is used by most policies for allocating organs while life expectancy has been increasingly valued^25,26^. In general, it is recommended to consider urgency and probability of success^26,27^. The main purpose of policies for allocating organs is to balance the justice-based system and the utility-based system.

However, achieving this balance is challenging as deceased donor PKT is a principle of dual effect^27,28^. It is a therapy that offers several benefits to the recipient, but also prolongs the waiting time on the list for patients who are already on dialysis, which might result in an increase in some of their risks, such as mortality.

Because of this ethical complexity, some authors suggest that deceased donor PKT should only be performed in places with high transplantation rates and reduced time on the waiting list^27,29^. Also, according to these authors, to be morally acceptable, preemptive transplantation needs to meet the following criteria^29^:

- the principal aim of the act, and the act itself, are good;
- the harmful effects are not intentionally pursued;
- the harmful effects are not the aim of the act and the good effect is not a direct cause-and-effect result of the harmful effect;
- the intended good effect is as great as or greater than the harmful effects and proportionate to them.

Indeed, ethical issues of PKT have been widely discussed in the last decades^24–27^. Because of these controversies, national transplant policies in some countries have restrictions that prevent a larger adoption of PKT worldwide. In Thailand, for example, PKT can only be performed in live kidney transplant recipients. In Spain, deceased donor PKT is usually available only after depletion of the waiting list^7^.

In Brazil, PKT can be legally performed not only for living donor kidney transplants but also for deceased donor kidney transplantation. According to the Ordinance 2600/2009, the in-state donation rate must be equal to or greater than the national average donation rate to allow deceased donor PKT within the respective Brazilian state. In Brazilian states where deceased donor PKT is permitted, the recipient must still meet one of the following criteria^30^:

- ≤ 18 years old and eGFR < 15 mL/min/1.73 m2 or;
- > 18 years old and eGFR < 10 mL/min/1.73 m^2^ or;
- Diabetic patients and eGFR < 15 mL/min/1.73 m^2^

Late referral to the nephrologist, which makes PKT not a possible treatment option for many ESKD patients, is another significant barrier. A careful clinical evaluation before kidney transplantation is mandatory, including not only comorbidities and possible contraindications, but also the patient’s lifestyle, past clinical history, family history, and associated risks. This assessment usually takes time, and when there is a late referral, the patient and transplant team do not have enough time to perform a careful PKT evaluation before renal replacement therapy is formally indicated.

Therefore, the patient and medical team must be aware of the path to be followed from diagnosis of chronic kidney disease (CKD) to the possibilities of choosing renal replacement therapies. It is also crucial that transplant centers are accessible to all patients and that their protocols are well clarified for professionals who assist these patients with CKD under conservative treatment.

We must consider that excluding PKT from therapeutic possibilities may mean depriving this patient of a valuable treatment alternative and all its advantages. Therefore, the first step is to invest in measures that address the main barriers, such as adequate education and training for patients and healthcare professionals. Table 1 summarizes the main barriers for PKT.

**Table 1.** Main issues and barriers for preemptive kidney transplantation

**Ethical issues**
Equity
Priority
Medical urgency
Efficiency
Utility
Therapeutic outcomes
Autonomy
Responsibility
**Late referral to the nephrologist**
**Accessibility to transplant centers to all patients**
**Long time requested to donor assessment**
**Prolonged time on the waiting list for patients on dialysis**

## When?

Due to the aforementioned benefits, one may believe that PKT should be performed as soon as clinically and ‘ethically’ possible. However, it is not easy to define the best moment for PKT. Although ‘early’ PKT could be theoretically desirable in order to maximize benefits for patients, we have to avoid initiating renal replacement therapy before irreversible kidney failure. A few studies have tried to answer this question and define the best moment to perform a PKT^31–33^.

A study evaluated 671 PKT (first and kidney-only) performed between 1984-2006 at two US centers and showed that higher pre-transplant kidney function was associated with better kidney function after transplantation. However, the difference in kidney function decreased over the first year. Higher allograft survival was not evidenced in recipients with higher pre-transplant GFR. Patients were divided in three groups based on pre-transplant kidney function estimated by MDRD equation: group 1: <10.0 mL/min/1.73 m^2^ (7.3 ± 1.7, N = 324), Group 2: 10.0–14.9 mL/min/1.73 m^2^ (12.0 ± 1.4, N = 217), and Group 3: ≥15.0 mL/min/1.73 m^2^ (21.1 ± 10.0, N = 130). This study concluded that PKT in the group with the most preserved GFR did not improve allograft survival after kidney transplantation compared to PKT with lower pre-transplant GFR^33^.

Studies have consistently failed to demonstrate advantages in allograft survival with ‘early’ PKT^31,32^. A study enrolling 19,471 PKT recipients between 1995 and 2009 from the United Network for Organ Sharing (UNOS) cohort evaluated patterns and implications of transplant timing in PKT. Again, this study did not find differences in patient survival or death-censored allograft survival between patients with different GFR at PKT – either in the entire cohort or in subgroup analyses of patients that could potentially benefit from ‘early’ PKT^34^.

It is worth noting that the studies mentioned above did not consider the benefits of not having the patient undergo maintenance dialysis. Patients who remain on dialysis for a long period have higher mortality than transplanted patients^35^.

Taken together, the studies suggest that ‘early’ PKT does not provide benefits to patients. In contrast, it might anticipate potential surgical risks to both recipients and donors. Therefore, the ideal time to perform PKT seems to be when patients have the lowest level of kidney function that keeps them without uremic or congestive signs and symptoms. Although preparation should indeed be started early, we must avoid performing a PKT when there is still a significant residual kidney function. This threshold is usually 15 mL/min/1.73 m^2^ for most patients, but the final decision must consider several factors, such as the clinical condition, the rate of decline of the native kidney function, and ethical issues. An individualized approach and a shared decision-making process whenever possible are highly recommended.

## How?

Kidney transplantation is superior to dialysis for most patients and provide better quality of life and survival advantages^35,36,37^. Therefore, kidney transplantation, preemptive or not, should always be encouraged, except when the procedure is contraindicated or refused. The patient should start its preparation from the moment of CKD diagnosis.

PKT has the advantage of eliminating risks associated with long-term dialysis^38^. Diabetics and children are often the most favored groups^32^. However, the indication for PKT should not be restricted to these patients.

Most policies of organ allocation accept the preemptive listing of patients with eGFR <15 mL/min/1.73 m^2^ if the irreversibility of kidney damage is confirmed^28^. Some studies suggest there is no benefit in transplanting a patient with eGFR >15 mL/min/1.73 m^2^, except in those who already have uremic signs and symptoms, which might occur among patients with diabetes^33^.

PKT can be performed with a living or a deceased donor, and preparation for PKT usually follows the same recommendations and protocols for non-PKT. The investigation of PKT candidates aims to identify conditions that increase the patient’s surgical risk and/or that may reduce the chances of the procedure’s success. It is important to carry out a careful anamnesis, investigating data such as family history and medical history. For patients at high risk, cardiovascular assessment is necessary. There is no consensus regarding the indication for pre-transplant catheterization. In general, it is recommended for high-risk patients, including those with diabetes and a history of ischemia^39^. Echocardiography is always indicated for suspected or confirmed cases of heart failure or valvular heart disease. For patients at increased risk of coronary artery disease, investigation of peripheral vascular disease through Doppler should be considered^39^.

Infectious diseases should also be part of the investigation while preparing potential PKT recipients. It is recommended that at least serology be performed to investigate for hepatitis B, hepatitis C, and HIV. Other serologies, such as cytomegalovirus and toxoplasmosis, are useful in post-transplant follow-up and are recommended by many transplant centers. In some countries, such as Brazil, these serologies are mandatory exams. Additional investigation to exclude arbovirus infection can be necessary in case of clinical suspicion, especially in endemic regions and during outbreaks^40^. Chest radiography should be ordered to investigate active infections or to detect, in combination with other exams, latent infections such as tuberculosis. Upon admission for transplantation, active acute infections must be investigated.

Simultaneous pancreas-kidney transplantation should be considered for diabetic patients^30^. Information regarding miscarriages in women or other events suggestive of coagulation abnormalities should not be ignored. Although they are not a barrier for transplantation, the existence of these pathologies changes pre-, intra-, or postoperatively management. The contraindications are summarized in Table 2.

**Table 2.** Absolute and relative contraindications for kidney transplantation

**Absolute Contraindications**
Reversible kidney failure
Active infections
Active malignancy
Documented treatment non-adherence
Uncontrolled psychiatric disease
Active substance abuse
Severe vasculopathy involving iliac arteries
Significantly shortened life expectancy^*^
**Relative Contraindications**
Blood transfusion in the last 15 days
Active peptic ulcer disease
Untreated coronary artery disease
Recent stroke history
Untreated viral hepatitis

* There is no consensus on the estimated minimum life expectancy. Some centers consider more than 1 year, others consider more than 5 years.

It is noteworthy that, although there are mandatory exams in the preparation of candidates for transplantation, patient assessment must be individualized and carried out according to comorbidities and risks. Transplantation centers usually have their own protocols, which include a list of exams to ensure safe investigation in compliance with local government regulations and policies, and consider the epidemiology of infections and structural difficulties of each service. Table 3 summarizes key points of pre-transplant assessment.

**Table 3.** Patient preparation for preemptive kidney transplantation

**Judicious Anamnesis**
Medical history, including previous surgeries and complications
Associated comorbidities, including psychiatric disorders
History of familiar disease
Etiology of original kidney disease and risk of recurrence after transplantation
History of immunizations
History of blood transfusions and miscarriages
Cardiovascular risk
**Physical Exam**
Body Mass Index (BMI)
Bilateral femoral and pedal pulses
**Laboratory Tests**
ABO typing
Complete blood count
Coagulogram
Fasting blood glucose
Cholesterol levels
Transaminases
**Complementary Tests**
Chest X-ray
Total abdominal ultrasound
Electrocardiogram
**Cardiovascular Assessment**
Evaluate echocardiogram indication, cardiac catheterization, vessel Doppler intra-abdominal or other
**Hematological Assessment**
Investigate history of miscarriages, venous thrombosis or other signs suggestive of coagulopathies
**Investigation of Acute, Chronic or Latent Infections**
Serologic testing for hepatitis B virus (HBsAg; HBsAb and HBcAb)
Serologic testing for hepatitis C virus
Serologic testing for HIV
Serologic testing for CMV
Serologic testing for syphilis
Tuberculosis testing (TST or IGRA)
Evaluate indication of other investigations according to local epidemiology such as toxoplasmosis, Chagas disease, HTLV, EBV and others
**Malignancy Investigation**
Investigate history and indications for cancer screening and evaluate contraindications

HBsAg: surface antigen; HBsAb: anti-surface antibody; HBcAb: anti-core antibody; HIV: human immunodeficiency virus; CMV: cytomegalovirus; HTLV: human T-cell lymphotropic virus; EBV: Epstein-Barr virus; TST: tuberculin skin test; IGRA: interferon-gamma release assay.

## Conclusion

PKT provides better post-transplant clinical outcomes, a better quality of life, and economic benefits than dialysis. However, the percentage of preemptive transplants performed annually worldwide remains low. There are several for this, such as ethical issues, late referral to the nephrologist, patient and medical team education, and a time-and energy-consuming donor evaluation process. Preparing patients for PKT is similar to non-PKT ones, but defining the right moment to perform it is not trivial. Avoiding PKT when there is still a significant residual kidney function is recommended as patients with higher GFR (>15 ml/min/1.73 m^2^) at the time of transplantation do not appear to have additional benefits. The nephrology community must encourage global initiatives to better understand the barriers and facilitate access to PKT.
